# Research on Physicochemical Properties and Taste of Coppa Influenced by Inoculation with *Staphylococcus* During Air-Drying Process

**DOI:** 10.3390/foods15030459

**Published:** 2026-01-28

**Authors:** Juanjuan Du, Linyuan Feng, Ying Wang, Jinxuan Cao, Jinpeng Wang, Yuemei Zhang, Xiaoyan Tang, Wei Wang, Yu Ding, Shuai Zhuang, Wendi Teng

**Affiliations:** 1 Key Laboratory of Geriatric Nutrition and Health, School of Food and Health, Beijing Technology and Business University, Beijing 100048, China; 2 Meat Processing Key Laboratory of Sichuan Province, College of Food and Biological Engineering, Chengdu University, Chengdu 610106, China; 3 Institute of Quality Standard & Testing Technology for Agro-Products, Key Laboratory of Agro-Product Quality and Safety, Chinese Academy of Agricultural Sciences, Beijing 100081, China; 4 Department of Food Science & Technology, Institute of Food Safety and Nutrition, Jinan University, Guangzhou 510632, China

**Keywords:** coppa, *Staphylococcus*, taste, metabolite profile, air-drying process

## Abstract

Air-dried pork coppa is highly favored for its unique organoleptic and flavor characteristics. However, the traditional long processing cycle and uncontrollable environmental conditions lead to unstable product quality. *Staphylococcus* mediates the reduction in nitrite to nitric oxide via nitrite reductase; the resulting nitric oxide then binds to myoglobin, forming nitrosylmyoglobin that endows meat products with a characteristic bright red. It could also improve the activity of lipase and protease, promoting the flavor. In this study, *Staphylococcus carnosus* and *Staphylococcus xylosus* as starter cultures have been applied to air-dried coppa. After *Staphylococcus* inoculation, the water activity and pH value of coppa significantly decreased compared with those of naturally fermented coppa (*p* < 0.05). Meanwhile, it improved the color and increased the hardness and chewiness, which in turn enhanced the overall taste of organoleptic acceptability. The ^1^H NMR spectra showed that the main taste metabolites were free amino acids and organic acids. Citrulline, formic acid, isobutyric acid, and isovaleric acid might be the key metabolites distinguishing between those with or without *Staphylococcus* inoculation. This study suggested that inoculation with *Staphylococcus xylosus* and *Staphylococcus carnosus* played an important role in improving the physicochemical properties and taste development of coppa.

## 1. Introduction

Air-dried pork coppa is a typical Italian dried-cured meat product, which is highly favored by consumers mainly for its unique organoleptic and flavor characteristics. The processing of air-dried coppa includes rubbing the curing agents, such as salts and spices, onto the surface of pork collar butts, then fermenting/air-drying with or without starter cultures [1]. Different chemical and physicochemical changes happen, showing great effects on the flavor quality of coppa [2]. With the globalization of meat consumption markets and the culinary culture exchanges, air-dried coppa has strong market prospects in eastern Asia, especially in China [3]. However, during the past few decades, there were many challenges to producing coppa in China due to different climates, geographical conditions, and so on. The low degree of industrialization, long processing cycle, and uncontrollable environmental conditions lead to unstable product quality [4].

The application of starter cultures in meat products has emerged as a promising strategy for improving food safety, optimizing flavor characteristics, and maintaining quality stability, which attracted extensive attention in recent years [5]. Among various starter cultures, *Staphylococci* are widely recognized for their role in fermented meat processing. *Staphylococcus* mediates the reduction in nitrite to nitric oxide via nitrite reductase. The resulting nitric oxide then binds to myoglobin, forming nitrosylmyoglobin that endows meat products with a characteristic bright red. It could also improve the activity of lipase and protease, promoting the flavor [5]. Specifically, *Staphylococcus carnosus* and *Staphylococcus xylosus* have been reported to offer great flavor potential for western sausages, such as Italian salami [6] and Spanish fermented sausages [7]. In China, Hu et al. found that fermented beef jerky with *Staphylococcus xylosus* as the starter culture possessed attractive color, odor, texture, taste, and overall acceptability [8]. However, up to now, no research has reported the application of *Staphylococcus carnosus* and *Staphylococcus xylosus* as starter cultures in coppa processing.

Taste is an important indicator of air-dried meat products, which largely determines consumers’ appetites and preferences [9]. Amino acids, peptides, organic acids, inorganic salts, and nucleotides were the main taste substances in coppa [2,10]. Studies have demonstrated that these taste compounds are mainly derived from protein degradation and lipid oxidation, which were the major contributors to texture parameters and taste attributes [11]. Nevertheless, a large accumulation of hydrolysis products did not necessarily serve to enhance sensory and taste characteristics in coppa [12], indicating that merely quantifying total hydrolysis products is insufficient to clarify taste development; instead, identifying the specific functional taste metabolites is essential. The specific effects of these two strains on coppa’s taste quality, as well as the underlying regulatory trajectories of taste-related metabolites induced by their inoculation, have not been elucidated to date.

Metabonomics, as a critical technological breakthrough, has become possible in small molecule separation and identification. Nuclear magnetic resonance spectroscopy (NMR), as a metabolomics technology, has unique advantages for the identification and quantification of small molecules. Compared with available analytical procedures, NMR not only simplifies sample preparation but reduces the time required for analysis [13]. Coupled with principal components analysis (PCA) and partial least squares discriminant analysis (PLS-DA), NMR-based metabolomics analysis has been used as a superior tool to analyze meat components, assess meat quality, and monitor diet metabonomic profiling [14]. Yang et al. systematically identified free amino acids, organic acids, nucleic acids, and small peptides in stewed pork hock in soy sauce by NMR [15]. Additionally, a metabolic insight was obtained on the formation of characteristic flavor of vinasse pike eel through ^1^H NMR spectroscopy [16]. These reports demonstrated that the relationship between metabolite profiling and meat quality could be revealed via NMR-based metabonomic strategy. However, few studies concentrate on comparing the components and profiles of taste substances using ^1^H NMR and further characterize their contributions to taste of coppa with *Staphylococcus carnosus* and *Staphylococcus xylosus* as starter cultures.

Herein, we hypothesized that inoculation with *Staphylococcus carnosus* and *Staphylococcus xylosus* would improve the physicochemical stability and sensory taste quality of coppa as well as regulate the profiles of taste-related metabolites. Based on these hypotheses, the aim of the present study was to assess the effects of *Staphylococcus carnosus* and *Staphylococcus xylosus* inoculation on the physicochemical properties and taste of coppa to verify the above directional expectations, characterize the components and profiles of taste substances using ^1^H NMR, and further discuss the contribution of key discriminant compounds to taste development while pursuing the exploratory goal of elucidating the metabolic mechanisms underlying the taste improvement induced by these starter cultures.

## 2. Materials and Methods

### 2.1. The Preparation of Staphylococcus and Sampling of Air-Dried Coppa

*Staphylococcus carnosus* (Number 337536) and *Staphylococcus xylosus* (Number 337469, North Na Biotech, Beijing, China) were separately inoculated into nutrient agar and nutrient broth, followed by shaking cultivation in an incubator at 37 °C for 24 h. After the strains grew, Gram staining was performed, and uniform morphological characteristics were observed under a microscope, indicating successful activation.

*Staphylococcus carnosus* and *Staphylococcus xylosus* were suspended in physiological saline, and the concentration of *Staphylococcus* cells was adjusted to 10^7^ CFU/mL by a spectrophotometer analysis (OD600) and refrigerated at 4 °C before inoculation. The verification of the total number of colonies was conducted using the 10-fold tandem dilution method and the plate counting method.

A total of 64 pieces of pork collar butts (average weight, 1.5 ± 0.3 kg) of domestic pigs (Landrace, conventional feeding, age 5–6 months, weight 80–90 kg) were purchased from a local slaughterhouse. Raw pork collar butts were trimmed to a similar size ((26 ± 1.5 cm) × (6 ± 1 cm) × (5 ± 1 cm)). The processing procedures were performed according to the previous studies with minor modifications [17]. Briefly, the mixture of 3.5% (*w*/*w*) NaCl, 0.01% (*w*/*w*) NaNO_2_, 0.3% (*w*/*w*) sugar, 0.15% (*w*/*w*) white pepper powder, 0.04% (*w*/*w*) nutmeg, and 0.1% (*w*/*w*) D-sodium erythorbate was uniformly rubbed on the surface of meat pieces, and then the meat pieces were dry-cured for 20 days, where RH kept at 85% and temperature controlled at 2 °C. After salting, the meat pieces were soaked in pre-cooled clean water at 2 °C for 5 min to wash away the excess salt and spices on the surface. These meat pieces were hung at 2 °C for 1 day for dehumidification and dehydration. A total of 16 samples were randomly selected from 64 samples, which were defined as the CK group. After sampling, these samples were regrouped together with the others for subsequent fermentation. The 64 meat pieces were randomly and evenly divided into four groups, with 16 pieces in each group. We then activated and prepared the bacterial solution to 10^7^ CFU/mL according to the above method. *Staphylococcus* cell suspensions, including *Staphylococcus carnosus*, *Staphylococcus xylosus*, or a 1:1 mixed *Staphylococcus carnosus* and *Staphylococcus xylosus*, were sprayed and inoculated on the surface of coppa samples at 1% of the meat weight to obtain an initial population of 10^6^ CFU/g. The coppa samples were divided into four groups: NA (with no *Staphylococcus*), RS (*Staphylococcus carnosus*), MS (*Staphylococcus xylosus*), and HS (*Staphylococcus carnosus* + *Staphylococcus xylosus*). The coppa samples were transferred to a fermentation room and underwent fermentation for 24 h at 30 °C, and further underwent air-drying for 40 days at 18 °C, 75% RH. Full fermentation of the meat pieces was achieved when aromatic compounds were released, and the product’s weight loss rate reached approximately 35%, after which the samples were vacuum-packaged and stored. During fermentation and air-drying, temperature and humidity inside the fermenter were regulated by its PLC to simulate an artificial microclimate. The refrigerant automatically adjusted the temperature changes, and the dehumidifier and the humidifier worked alternately to ensure stable humidity. The fermentation and air-drying status is observed three times a day at 8 a.m., 2 p.m., and 8 p.m., and records are kept. Take three complete coppa at different times (0, 10, 20, 30, and 40 days) of the air-drying process, respectively, and cut into small pieces (5 cm × 5 cm × 5 cm). They were then packaged in tin foil and stored at −20 °C for analysis.

### 2.2. Physicochemical Analysis of Coppa During the Air-Drying Process

#### 2.2.1. Water Activity Measurement

Water activity of the samples was measured using the AquaLab CX-2 water activity equipment (Decagon Devices, Pullman, WA, USA) and the temperature was kept at 25 °C.

#### 2.2.2. pH Value Analysis

All the samples were cut into 5 × 5 × 2 mm cubes. The pH meter (Testo 205, Titisee-Neustadt, Germany) was first calibrated by three standard solutions, and then the probe was inserted directly into the meat samples to a depth above the probe.

#### 2.2.3. Color Evaluation

All the samples were cut into 2 × 2 × 2 cm cubes with smooth and flat surfaces. Color was analyzed using a colorimeter (Konica Minolta, Tokyo, Japan) with illuminant D65, observer angle of 10°, and aperture size of 8.0 mm. Nine different focuses of each coppa were randomly taken for the evaluation of CIE color parameters [lightness (L*, light/dark), redness (a*, red/green), and yellowness (b*, yellow/blue)].

#### 2.2.4. Texture Analysis

The measurement of hardness, springiness, cohesiveness, and chewiness was performed by texture analyzer (TA-XT Plus; Stable Micro Systems, Godalming, UK) equipped the special probe (P 50), as described by Xu et al. [18]. Hardness (g) was expressed peak force of the first compression cycle; springiness was calculated as the height that the myofibril fibers recover during the time elapsing between the two compression cycles; cohesiveness was calculated as the ratio of the area under the second curve to the area under the first curve; chewiness was expressed as the multiplication of hardness, cohesiveness, and springiness.

The sample was cut into cubes of 2 mm in length, width, and height. A P50 (d = 50 mm) probe was used. The parameters were set at a pre-measurement rate of 5.0 mm/s, a test rate of 2 mm/s, a post-measurement rate of 5 mm/s, a compression rate of 40%, and a pressure of 5.0 g. The four indicators of the sample were tested: hardness, springiness, cohesiveness, and chewiness. Three parallel determinations were conducted on each group of meat samples, and each parallel was repeated three times.

### 2.3. Sensory Evaluation

The sensory analysis was performed according to the description of Yao et al. with some modifications [19]. The sensory attributes, including color, juiciness, taste (overall taste, saltiness, umami, sweetness, bitterness, sourness, and aftertaste), smell, and texture, were scored by the trained panel consisting of five males and five females (age 25–45) with rich experience. The laboratory was equipped with independent evaluation booths to avoid mutual interference between assessors, and the environmental conditions were strictly controlled: the room temperature was maintained at 23 ± 2 °C, relative humidity at 50 ± 5%.

Before evaluating the sensory attributes of samples, all 10 assessors were trained and qualified in accordance with the national standard of the People’s Republic of China GB/T 16291.1 [20] and ISO 8586-1:2012 [21] (sensory analysis—general guidance for the selection, training and monitoring of assessors) to ensure that the score description deviation among the group members was less than 1. This approach helps to enhance the accuracy and reliability of the evaluation results. In addition, the coefficient of variation (CV) of each assessor’s repeated scores for the same sample (each sample was encoded and re-evaluated after a 24 h interval) was significantly lower than the 10% threshold recommended for acceptable repeatability in sensory analysis. We also conducted a triangle test prior to formal evaluation, using samples with known taste differences. The panel achieved a correct discrimination rate, which exceeds the 75% benchmark for a panel with satisfactory discrimination ability according to ISO 4120:2004 [22]. At the same time, references on the strength of sensory attributes were provided to help the score.

During the evaluation process, a strictly defined chewing scheme was implemented consistently by all assessors. Each coppa treatment group at day 40 had three independent experimental replicates, respectively. To alleviate the sensory fatigue of the assessors, one coppa was selected from each group at a time for sensory evaluation after a 24 h interval, with a total of three batches. Each tasting session will be repeated twice to ensure accuracy and consistency in the evaluation process. The coppa samples were cut into slices (1 mm thick, cut using a precision slicer, randomly labeled with a code to avoid assessor bias) and chewed 15 times with uniform chewing force, and sensory attributes were scored immediately after chewing. The assessment should be controlled within 3 min after cutting. Assessors rinsed their mouths with distilled water (at 23 ± 2 °C) for 10 s before evaluating each sample to eliminate residual taste interference. The scoring intensity ranges from 1 to 10 points. The scoring criteria are shown in Supplementary Tables S1 and S2.

### 2.4. Metabolite Extraction

The extraction of metabolites was performed according to our previous study with minor modifications [14]. For each treatment group (CK, NA, HS), we prepared nine independent coppa pieces as biological replicates, corresponding to three batches of coppa production (three pieces per batch). Briefly, the pieces were thawed at 4 °C for 30 min. The 50 mg of samples were homogenized twice in an ice water bath with 1 mL of methanol/water (2/1, *v*/*v*, pre-cooled to −20 °C) at a speed of 5000 r/min for 3 min. The homogenate was centrifuged at 12,000× *g* for 15 min at 4 °C. The supernatants were further freeze-dried at −50 °C under 0.01 mbar for 72 h. The extraction was re-dissolved in 600 μL of pre-cooled phosphate buffer (containing 0.005% TSP, 50% D_2_O and 0.1% NaN_3_, pH 7.4, 4 °C) and then centrifuged at 12,000× *g* for 10 min at 4 °C. The 550 μL of the supernatants were transferred into an NMR tube for subsequent analysis.

### 2.5. NMR Analysis

The ^1^H NMR spectra of all the extracts were performed at 25 °C using a Bruker Avance 600 MHz Spectrometer (Bruker Biospin, Rheinstetten, Germany) with an ultra-low temperature detection probe at the operating condition of 600.13 MHz. The metabolite profile of each sample was achieved via a standard Bruker pulse sequence (NOESYGPPR1D, Bruker library, Rheinstetten, Germany, RD-90°-t1–90°-tm-90°-acquisition), with 100 ms mixing time and a weak irradiation during recycle delay (RD, 1 s) to suppress the solvent signal. A 90° pulse length was adjusted to 15 μs and t1 to 2 μs. A total of 32 transients were gained with a 20 ppm spectral width into 32 K data points. The individual metabolite was assigned according to the metabolome database of The Metabolomics Innovation Centre.

We import the ^1^H NMR offline data into the Chenomx NMR suite (version 8.5) software to calibrate the phase, baseline, and calibration (DSS peak at 0.0 ppm), and adjust the spectral peaks. Based on the concentration and peak area of DSS as the standard, and combined with the built-in database of Chenomx, we fit and compare the signal peaks one by one for qualitative analysis. We quantitatively determine according to the proportional relationship between peak area and substance concentration, obtaining metabolites and absolute concentration values. Then, we export it into a table to obtain the variable matrix of the sample. Spectral data were first normalized using the probabilistic quotient normalization (PQN) method. Normalized spectral data were further subjected to unit variance scaling (UV-scaling) to standardize each variable (metabolite resonance peak) with a mean of 0 and a variance of 1, which eliminates the bias caused by metabolites with extremely high or low concentrations. After normalizing the exported data, we conducted subsequent PCA and PLS-DA analyses.

### 2.6. Statistical Analysis

All the experiments were carried out at least in triplicate. Data are shown as means ± standard deviations (SD). Statistical differences (*p* < 0.05) were evaluated via one-way ANOVA analysis, followed by Duncan’s multiple-comparison test using software SPSS 26.0 (IBM Inc., Chicago, IL, USA). Sensory evaluation was analyzed via two-way ANOVA. PCA was carried out to characterize the components and contents of metabolites. PLS-DA was performed to further analyze the key taste compounds using SIMCA 14.0. Both PCA and PLS-DA were conducted based on the normalized and scaled data matrix, with PLS-DA further validated via CV-ANOVA to avoid overfitting.

## 3. Results

### 3.1. The Effects of Staphylococcus Inoculation on the Water Activity of Coppa

**Figure 1:**
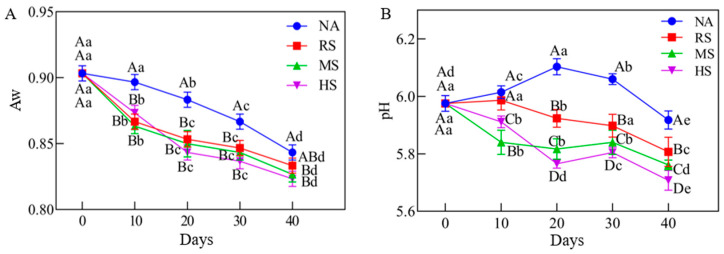
The changes of water activity (**A**) and pH values (**B**) of coppa during air-drying process. ^a–e^: different letters indicate significant differences in different stages of air-drying with the same treatment (*p* < 0.05). ^A–D^: different letters indicate significant differences between different treatment groups at the same stage of air-drying (*p* < 0.05).

The reduction in water activity can effectively extend the shelf life and improve the product safety. Overall, regardless of the *Staphylococcus* inoculation, the water activity values decreased over time (Figure 1A), but the decrease rate varied. In the NA group, the water activity slowly decreased from 0.9 ± 0.01 to 0.84 ± 0.01 at day 40. However, at day 30, the water activity in the other three inoculation (RS, MS, HS) groups rapidly decreased to 0.85 ± 0.01, 0.84 ± 0.01, and 0.84 ± 0.01, respectively. Also at day 30, we observed that there was no significant difference among the inoculation groups, while a significant difference compared with the NA group. During the air-drying process, the decrease in water activity was mainly caused by water evaporation and protein denaturation. On one hand, the water on the surface of the meat product slowly evaporated, resulting in a decrease in water activity. On the other hand, when the acidic denaturation of myofibrillar protein occurred, its water-holding capacity weakened, leading to reduced water activity. These results suggested that inoculation with *Staphylococcus carnosus* and *Staphylococcus xylosus* could faster reduce the water activity of air-dried coppa, which could inhibit the multiplication of spoilage and pathogenic microorganisms, thus ensuring the safety and quality of meat products [23].

### 3.2. The Effects of Staphylococcus Inoculation on the pH Value of Coppa

pH is one of the most important indicators, which can reflect the accumulation of organic acids and alkaloids. As shown in Figure 1B, the initial pH of all samples was around 6.0, followed by a decrease in pH across the four treatment groups, with substantial variation in the rate of decline among groups. The pH value in the NA group first increased and then decreased. However, as the *Staphylococci* dramatically reproduced in the early stage of air-drying, the pH values in the RS, MS, and HS groups markedly decreased. During the air-drying of 20–30 days, the pH of the inoculation groups showed a slight increase. At the end of air-drying, the HS group had the lowest pH value, followed by the MS, RS, and NA groups. The three inoculation groups showed a significantly lower pH value than the NA group. It indicated that inoculation with *Staphylococcus* significantly reduced the pH value and the water activity of coppa, which was beneficial for inhibiting the growth of pathogenic microorganisms.

### 3.3. The Effects of Staphylococcus Inoculation on the Color of Coppa

Another important characteristic linked to the meat quality and able to importantly affect the consumers’ acceptance is the color [24]. Throughout the air-drying process, the lightness values (L*) in the four groups significantly reduced (Table 1), which might be due to water loss on the surface of the meat weakening the ability of light reflection, so that the meat gradually turned into dark red. The L* values were compared among the four groups at 0 and 10 days. The results showed that there were no statistically significant differences among the four groups. At day 20, there was a significant difference between the HS group and the NA group. At day 40, the HS group had the highest L* value, followed by the MS, RS, and NA groups. The L* values in the HS group and MS group were significantly higher than in the NA group. There was no significant difference among the RS, MS, and HS groups.

The redness values (a*) of the three inoculation groups increased within 10 days. Studies have reported that during the early stage of fermentation and drying, *Staphylococcus* multiplied in large numbers, causing nitrite to be reduced by nitrite reductase to nitroso. This nitroso combined with myoglobin to form nitrosomyoglobin, resulting in the meat taking on a rose-red color [25]. But at the late stage of air-drying, the decreased pH inhibited the growth of *Staphylococcus* and darkened the color. There was no significant difference for a* values in the three inoculation groups at day 40. Particularly, only the HS group showed no significant difference for the a* values between day 0 and day 40, indicating that the mixed *Staphylococcus carnosus* and *Staphylococcus xylosus* could act as a color protectant. Nevertheless, the a* value of the NA group showed a decreasing trend throughout the air-drying process, from 11.74 to 8.8, which was significantly lower than that in the other three groups.

The yellowness values (b*) of NA and RS groups showed a significant decrease throughout the air-drying process, whereas there was no significant difference between day 0 and day 40 for MS and HS groups. At day 40, the b* values of the HS and MS groups were significantly higher than those of the NA group, and there was no significant difference among the RS, MS, and HS groups. The above results indicated that co-inoculation with *Staphylococcus carnosus* and *Staphylococcus xylosus* played a positive role in improving the color of air-dried pork coppa and working up consumers’ acceptance.

### 3.4. The Effects of Staphylococcus Inoculation on the Texture of Coppa

Table 2 shows the differences in the hardness, springiness, cohesiveness, and chewiness of coppa. The results revealed that at the end of air-drying, hardness and chewiness of the three inoculation groups were significantly higher than those of the NA group, especially the HS group. This might contribute to the fact that inoculation with *Staphylococcus* promoted water loss, causing the muscle fibers to become firmer. The above results indicated that inoculation with *Staphylococcus carnosus* and *Staphylococcus xylosus* could moderately increase the chewiness and hardness of coppa, which enriched the pleasurable sensation of chewing.

### 3.5. The Effects of Staphylococcus Inoculation on the Taste Sensory Evaluation of Coppa

**Figure 2:**
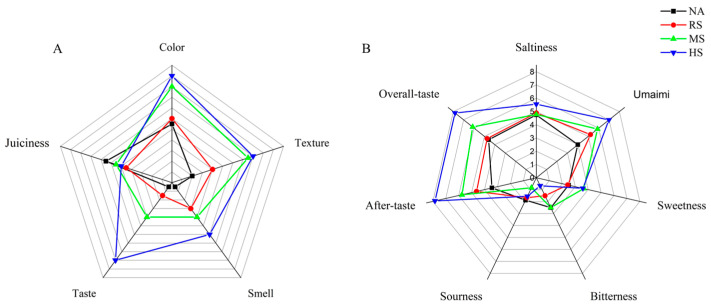
Sensory evaluation analysis of coppa at day 40. (**A**) Overall sensory evaluation; (**B**) Taste sensory evaluation.

Sensory characteristics are shown in Figure 2. Taste, a sensory attribute that requires subjective human perception to reflect the integrated effects of taste-active metabolites, was the indicator with the greatest difference among the four groups (Figure 2A). The radar chart showed that the overall taste score of the HS group was the highest, 7.83 ± 0.41, while that of the NA, RS, and MS groups was 4.6 ± 0.55, 4.75 ± 0.71, and 6.14 ± 0.69, respectively. In addition, the intensity of aftertaste and umami in the HS group was also higher than that in the other three groups, from largest to smallest, which were the HS group (7.83 ± 0.41, 7.0 ± 0.5), MS (5.75 ± 0.46, 5.9 ± 0.57), RS (4.63 ± 0.52, 5.22 ± 0.97), and NA (3.43 ± 0.53, 4.0 ± 1.0) groups. As for the sourness, the score of the HS group (1.56 ± 0.53) was lower than that of the NA group (1.88 ± 0.64). The HS group also had a moderate sourness and the lowest bitterness, which showed the highest acceptability. These results indicated that inoculation with *Staphylococcus carnosus* and *Staphylococcus xylosus* could bring more favorable taste attributes to coppa. Given that the quality traits (water activity, pH dynamics, color, texture, and sensory acceptability) of the HS group were significantly superior to those of the two single-strain groups, we focused on analyzing the impact of the co-fermentation of *Staphylococcus carnosus* and *Staphylococcus xylosus* on taste metabolites.

### 3.6. Metabolite Profile Analysis of Coppa

**Figure 3:**
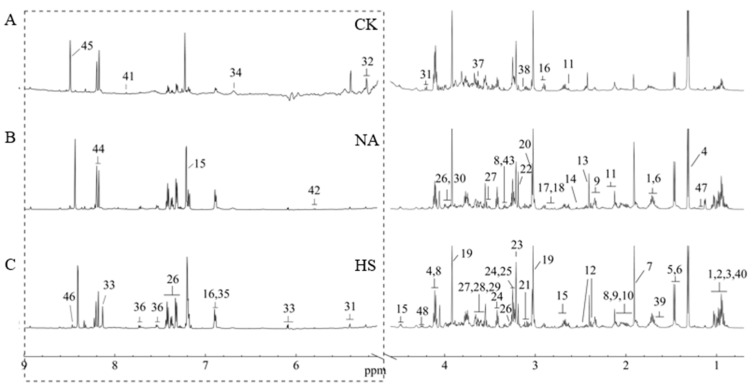
Representative ^1^H NMR spectra of coppa. The dotted region was vertically magnified 8 times. Keys: 1: leucine; 2: isoleucine; 3: valine; 4: lactic acid; 5: alanine; 6: lysine; 7: acetic acid; 8: proline; 9: glutamic acid; 10: glutamine; 11: methionine; 12: pyroglutamic acid; 13: succinate; 14: β-alanine; 15: anserine; 16: tyramine; 17: aspartic acid; 18: asparagine; 19: creatine; 20: creatinine; 21: niacin; 22: choline; 23: glycerophosphorylcholine; 24: taurine; 25: betaine; 26: phenylalanine; 27: inositol; 28: isovaleric acid; 29: threonine; 30: serine; 31: sucrose; 32: glucose; 33: inosine; 34: fumaric acid; 35: tyrosine; 36: tryptophan; 37: glycine; 38: citrulline; 39: 2-hydroxybutyric acid; 40: isobutyric acid; 41: xanthine nucleoside; 42: uracil; 43: residual methanol; 44: hypoxanthine; 45: formic acid; 46: 5′-inosinic acid; 47: ethanol; 48: fructose. (**A**) Representative ^1^H NMR spectra of coppa samples before fermentation; (**B**) Representative ^1^H NMR spectra of coppa samples after fermentation with no *Staphylococcus*); (**C**) Representative ^1^H NMR spectra of coppa samples after fermentation with *Staphylococcus carnosus* and *Staphylococcus xylosus*.

For the metabolite profile analysis of coppa, we compared the taste differences with or without *Staphylococcus* inoculation at day 40. At the same time, dry-cured pork collar butts that did not undergo fermentation and air-drying were used as control (CK group). Herein, the ^1^H NMR spectra of coppa in the three groups are shown in Figure 3. A total of 48 taste substances were identified, including 24 amino acids and their derivatives (alanine, asparagine, aspartic acid, betaine, citrulline, glutamic acid, glutamine, glycine, isoleucine, leucine, lysine, methionine, phenylalanine, proline, serine, taurine, threonine, tryptophan, tyrosine, valine, β-alanine, anserine, pyroglutamate, tyramine), nine organic acids (2-hydroxybutyric acid, acetic acid, formic acid, fumaric acid, isobutyric acid, isovaleric acid, lactic acid, succinic acid, and nicotinic acid), three sugars (fructose, glucose, and sucrose), five nucleic acids and their derivatives (hypoxanthine, 5′-inosinic acid, inosine, uracil, and xanthosine), three alcohols (methanol, ethanol, myo-inositol), two phospholipid derivatives (choline, glycerophosphorylcholine), two guanidino compounds (creatine, creatinine), and one organic amine (Tyramine).

**Figure 4:**
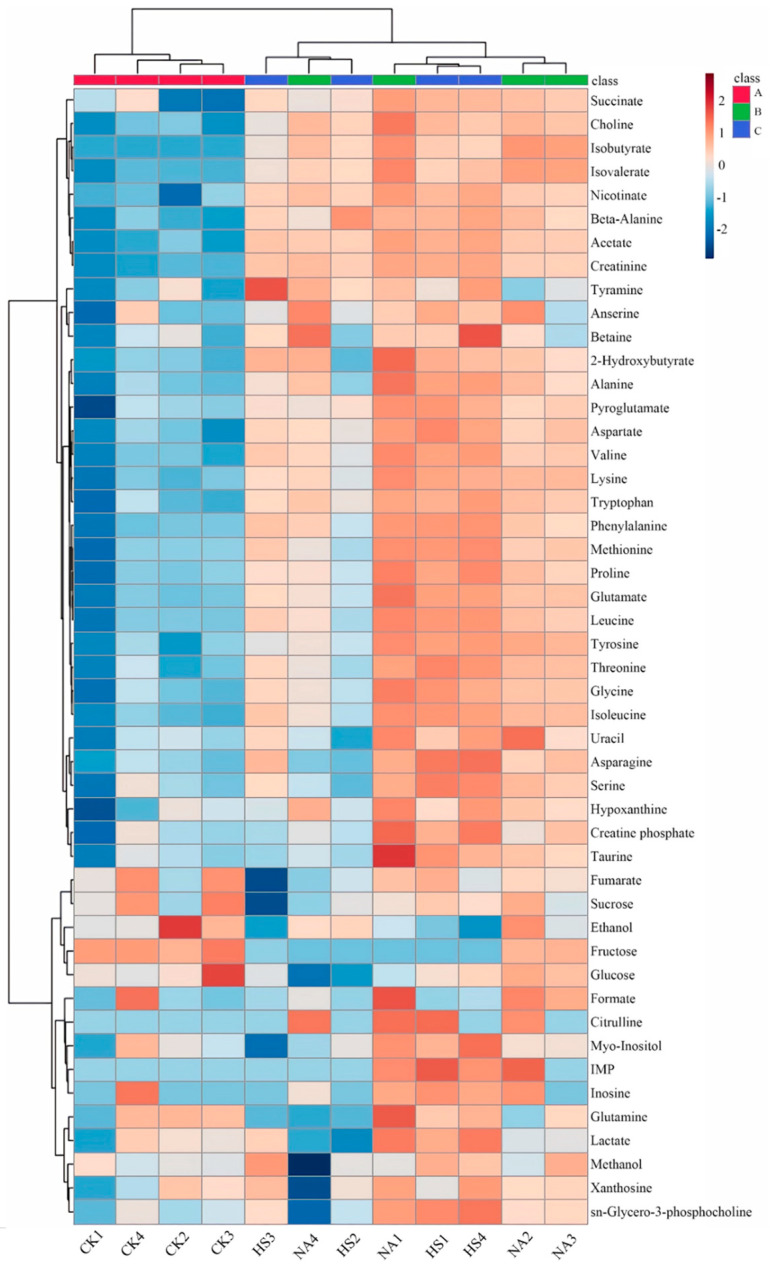
Hierarchical cluster analysis heatmap on the metabolites at day 40.

To compare the changes of each metabolite, hierarchical cluster analysis heatmap (Figure 4) was performed to explore the information of metabolite profiles, where each row represented a metabolite and each column represented a coppa sample. According to the information in the heat map, it could be found that the taste profile in the CK group was significantly different from the other two groups, and the taste substances in the NA group and HS group were mainly free amino acids and organic acids.

**Figure 5:**
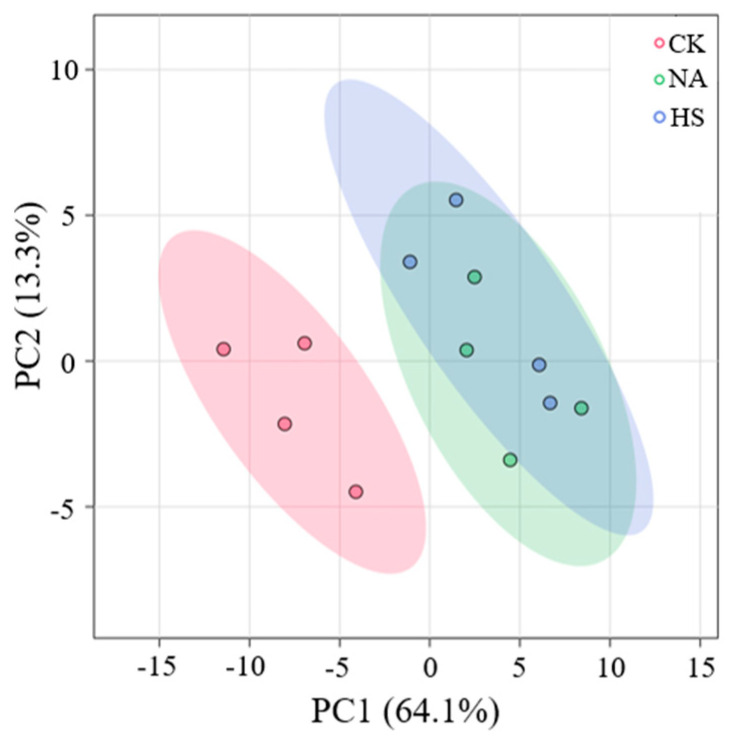
PCA analysis on the metabolites at day 40.

Furthermore, PCA was employed to further characterize the differences in the metabolite profile. The ellipses represented the 95% confidence intervals for each group. According to Figure 5, the PCA score plot showed that 77.4% of the variability was explained by the first two principal components, accounting for 64.1% and 13.3% of the total variance, respectively, and PC1 values increased during the air-drying process. The samples in the CK group were located on the negative side of the PC1 values, while the samples in the NA group and the HS group were located on the positive side of the PC1, indicating a significant difference. Overall, these results indicated that the taste metabolites significantly changed during the air-drying process, but the taste profiles in the NA group and HS group were relatively close.

### 3.7. Metabolite Composition Analysis and Their Contributions to Taste Development

**Figure 6:**
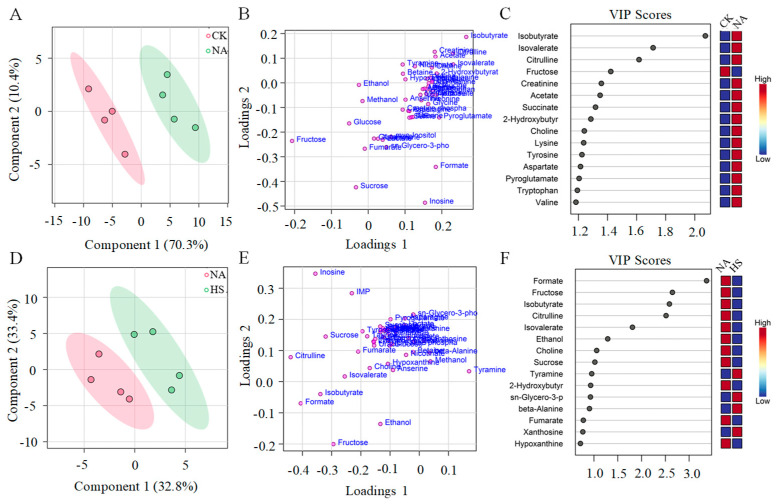
PLS-DA score plot (**A**,**D**), PLS-DA loading plot (**B**,**E**), and VIP scores (**C**,**F**).

To further obtain more information on the taste, the similarity or difference in metabolites and taste scores between samples was conducted by PLS-DA. The model achieved a CV-ANOVA *p* value < 0.001, which confirmed that the model had a statistically significant predictive ability and that the observed separations between two groups (CK and NA, NA and HS) were statistically significant, rather than arising from random variation. This metric was complemented by the satisfactory fitting and predictive performance (R^2^X = 0.851, R^2^Y = 0.924, and Q^2^ = 0.858). The permutation tests confirmed that the model was not overfitted, further validating the robustness of the PLS-DA model. As shown in Figure 6A, the samples in the CK group and the NA group could be significantly separated. In the PLS-DA loadings plot, the farther the location of a metabolite was from the origin of the axes, the greater its contribution to the differences between the groups. As shown in Figure 6B, the metabolites, such as isobutyrate, isovalerate, citrulline, and fructose, made a significant contribution to the differentiation between the two groups, indicating that these metabolites changed significantly during the air-drying process. Similarly, the separation of the NA group and the HS group could be seen in Figure 6D. Multiple metabolites, such as formate, fructose, isobutyrate, citrulline, and isovalerate, functioned as important contributions to the taste differentiation with or without *Staphylococca* inoculation (Figure 6E).

In addition, the variable importance of the projection plots (VIP) was employed to evaluate the contribution of metabolites towards influencing the taste (Figure 6C). Fifteen metabolites, including amino acids and their derivatives (aspartic acid, tyrosine, tryptophan, valine, citrulline, lysine, pyroglutamic acid), organic acids (succinic, acetic, isovaleric, 2-hydroxybutyric, and isobutyric), ammonium-based compounds (choline), sugar (fructose) and organic amines (creatinine), showed a high VIP value (more than 1), indicating that these metabolites could have a key contribution to taste differences between the CK group and the NA group. The metabolites of the NA group changed significantly compared with the CK group during the fermentation process, and the concentrations of most metabolites showed an increasing trend (except formic acid, fumaric acid, hypoxanthine, and tyramine xanthine). As could be seen from Figure 6F, organic acids (isobutyric acid, 2-hydroxybutyric acid, fumaric acid, isovaleric acid, formic acid), sugars (fructose, sucrose), amino acids and their derivatives (tyramine, β-alanine, citrulline), nucleic acids and their derivatives (hypoxanthine, xanthine nucleoside), and ammonium-based compounds (choline) played a significant role in influencing the taste differences between the NA group and the HS group. Among them, the concentrations of several metabolites, namely isobutyric acid, isovaleric acid, formic acid, and citrulline in the NA group were significantly higher than those in the HS group.

To further evaluate the contribution of the key taste substances with or without *Staphylococca* inoculation, the contents of 15 key metabolites (isobutyric acid, 2-hydroxybutyric acid, fumaric acid, isovaleric acid, formic acid, fructose, sucrose, xanthine riboside, hypoxanthine, ethanol, glycero phosphorylcholine, choline, tyrosine, and β-alanine) were further analyzed. Data in Table 3 revealed that the contents of citrulline, formic acid, isobutyric acid, and isovaleric acid in the HS group were significantly lower than those in the NA group, indicating that the four metabolites might be important taste differentiators. Citrulline contributed sweet, sour, salty, and umami flavors at different concentrations, respectively [26], while formic acid imparted a sour taste [27], and isobutyric acid and isovaleric acid contributed to a smelly-sour taste [28]. The reduction in the contents of these four organic acids might be an important reason why the sourness of the air-dried coppa after inoculation with *Staphylococcus carnosus* and *Staphylococcus xylosus* in the HS group was lower than that in the CK group in the sensory evaluation results.

## 4. Conclusions

This study systematically investigated the effects of co-inoculation with *Staphylococcus carnosus* and *S. xylosus* on the processing quality and taste development of air-dried pork coppa, with integrated analyses of physicochemical properties, dynamic microbial communities, ^1^H NMR-based metabolomics, and sensory evaluation. During the processing of air-dried coppa, co-inoculation with *Staphylococcus carnosus* and *Staphylococcus xylosus* significantly reduced the water activity and pH value of coppa, improved the color stability, and improved the texture by increasing the hardness and chewiness. The ^1^H NMR spectra identified that the main taste metabolites of coppa were free amino acids and organic acids. Citrulline, formic acid, isobutyric acid, and isovaleric acid might be the key metabolites distinguishing between those with or without *Staphylococcus* inoculation. This study suggested that inoculation with *Staphylococcus carnosus* and *Staphylococcus xylosus* played an important role in improving the physiochemical properties and taste development of air-dried pork coppa.

## Figures and Tables

**Table 1:** The changes of L*, a*, b* values of coppa during air-drying process.

	Time (Days)	NA	RS	MS	HS
L*	0	57.67 ± 0.86 ^Aa^	57.67 ± 0.86 ^Aa^	57.67 ± 0.86 ^Aa^	57.67 ± 0.86 ^Aa^
	10	52.50 ± 2.85 ^Ab^	53.13 ± 0.54 ^Ab^	54.08 ± 1.09 ^Ab^	55.24 ± 0.82 ^Ab^
	20	48.20 ± 2.46 ^Bc^	49.35 ± 0.62 ^Bc^	50.31 ± 0.28 ^ABc^	52.29 ± 0.62 ^Ac^
	30	38.97 ± 1.27 ^Bd^	39.38 ± 0.53 ^Bd^	40.29 ± 0.51 ^ABd^	41.08 ± 0.62 ^Ad^
	40	37.92 ± 0.74 ^Bd^	38.32 ± 0.46 ^ABd^	39.13 ± 0.22 ^Ad^	39.20 ± 0.41 ^Ae^
a*	0	11.74 ± 0.61 ^Aa^	11.74 ± 0.61 ^Aa^	11.74 ± 0.61 ^Aa^	11.74 ± 0.61 ^Aab^
	10	10.88 ± 0.50 ^Ba^	12.45 ± 0.33 ^Aa^	12.69 ± 0.04 ^Aa^	13.05 ± 0.21 ^Aa^
	20	9.66 ± 0.50 ^Bb^	11.78 ± 0.69 ^Aa^	12.15 ± 0.39 ^Aa^	12.34 ± 0.60 ^Aab^
	30	9.29 ± 0.44 ^Bb^	10.65 ± 0.47 ^ABb^	10.12 ± 0.69 ^ABc^	11.23 ± 1.19 ^Ab^
	40	8.80 ± 0.39 ^Bb^	10.42 ± 0.37 ^Ab^	10.77 ± 1.12 ^Abc^	11.01 ± 0.71 ^Ab^
b*	0	11.45 ± 0.83 ^Aa^	11.45 ± 0.83 ^Aa^	11.45 ± 0.83 ^Aab^	11.45 ± 0.83 ^Aa^
	10	10.69 ± 0.97 ^Aab^	10.46 ± 0.74 ^Aab^	12.02 ± 0.91 ^Aa^	12.37 ± 1.61 ^Aa^
	20	9.71 ± 1.02 ^Bbc^	9.69 ± 0.06 ^Bb^	11.30 ± 0.61 ^Aab^	11.84 ± 0.08 ^Aa^
	30	9.40 ± 0.55 ^Bbc^	9.58 ± 0.31 ^Bb^	10.66 ± 0.60 ^Aab^	11.04 ± 0.69 ^Aa^
	40	8.75 ± 0.36 ^Bc^	9.93 ± 0.99 ^ABb^	10.20 ± 0.65 ^Ab^	10.86 ± 0.43 ^Aa^

^a–e^: different letters indicate significant differences in different fermentation times of air-drying with the same treatment (*p* < 0.05). ^A,B^: different letters indicate significant differences between different treatment groups at the same fermentation time of air-drying (*p* < 0.05).

**Table 2:** The changes of texture properties of coppa during air-drying process.

	Time (Days)	NA	RS	MS	HS
Hardness (g)	0	269.39 ± 109.15 ^Ab^	269.39 ± 109.15 ^Ab^	269.39 ± 109.15 ^Ac^	269.39 ± 109.15 ^Ac^
	10	395.14 ± 176.07 ^Bab^	291.51 ± 20.06 ^Bb^	747.85 ± 111.48 ^Abc^	234.62 ± 94.02 ^Bc^
	20	686.53 ± 63.75 ^BCab^	323.23 ± 61.33 ^Cb^	982.53 ± 373.30 ^Bbc^	1606.43 ± 481.26 ^Ab^
	30	712.75 ± 99.37 ^Bab^	540.22 ± 224.60 ^Bb^	1190.49 ± 845.38 ^ABab^	1978.77 ± 325.10 ^Aab^
	40	796.83 ± 413.30 ^Ca^	1588.83 ± 265.17 ^Ba^	1904.28 ± 88.99 ^ABa^	2260.84 ± 398.98 ^Aa^
Springiness	0	0.20 ± 0.01 ^Aa^	0.20 ± 0.01 ^Ab^	0.20 ± 0.01 ^Aa^	0.20 ± 0.01 ^Aa^
	10	0.22 ± 0.02 ^Aa^	0.23 ± 0.01 ^Aa^	0.22 ± 0.01 ^Aa^	0.23 ± 0.02 ^Aa^
	20	0.21 ± 0.00 ^Aa^	0.22 ± 0.01 ^Aab^	0.22 ± 0.00 ^Aa^	0.21 ± 0.01 ^Aa^
	30	0.22 ± 0.02 ^Aa^	0.20 ± 0.02 ^Ac^	0.22 ± 0.01 ^Aa^	0.21 ± 0.01 ^Aa^
	40	0.20 ± 0.01 ^Aa^	0.19 ± 0.02 ^Abc^	0.22 ± 0.02 ^Aa^	0.20 ± 0.03 ^Aa^
Cohesiveness	0	0.23 ± 0.00 ^Aab^	0.23 ± 0.00 ^Aab^	0.23 ± 0.00 ^Aa^	0.23 ± 0.00 ^Ab^
	10	0.21 ± 0.02 ^Bc^	0.25 ± 0.00 ^Aa^	0.26 ± 0.01 ^Aa^	0.27 ± 0.01 ^Aa^
	20	0.23 ± 0.00 ^ABab^	0.25 ± 0.01 ^Aa^	0.25 ± 0.01 ^Aa^	0.23 ± 0.02 ^Bb^
	30	0.24 ± 0.02 ^ABa^	0.22 ± 0.01 ^Bb^	0.25 ± 0.02 ^Aa^	0.25 ± 0.02 ^Aab^
	40	0.24 ± 0.02 ^Aa^	0.21 ± 0.03 ^Ab^	0.25 ± 0.03 ^Aa^	0.25 ± 0.03 ^Aab^
Chewiness	0	12.83 ± 5.88 ^Ab^	12.83 ± 5.88 ^Ab^	12.83 ± 5.88 ^Ac^	12.83 ± 5.88 ^Ab^
	10	17.93 ± 9.45 ^Bab^	16.84 ± 1.72 ^Bb^	41.90 ± 8.94 ^Abc^	14.65 ± 4.55 ^Bb^
	20	33.41 ± 2.70 ^BCa^	17.29 ± 2.91 ^Cb^	52.43 ± 19.24 ^ABabc^	74.40 ± 20.12 ^Aa^
	30	21.70 ± 11.89 ^Bab^	25.02 ± 2.37 ^Bb^	66.40 ± 51.10 ^ABab^	108.28 ± 27.73 ^Aa^
	40	37.41 ± 16.47 ^Ba^	65.60 ± 26.63 ^ABa^	102.66 ± 23.51 ^Aa^	118.57 ± 52.29 ^Aa^

^a–c^: different letters indicate significant differences in different fermentation times of air-drying with the same treatment (*p* < 0.05). ^A–C^: different letters indicate significant differences between different treatment groups at the same fermentation time of air-drying (*p* < 0.05).

**Table 3:** The content changes of key taste compounds of coppa.

Metabolites	Average ± SD (mg/g)		
	CK	NA	HS
2-hydroxybutyric acid	0.022 ± 0.007 ^b^	0.152 ± 0.089 ^a^	0.106 ± 0.058 ^ab^
Choline	0.051 ± 0.017 ^b^	0.277 ± 0.086 ^a^	0.195 ± 0.044 ^a^
Citrulline	ND	0.090 ± 0.069 ^a^	ND
Ethanol	0.030 ± 0.012 ^a^	0.025 ± 0.007 ^a^	0.016 ± 0.007 ^a^
Formic acid	0.034 ± 0.025 ^b^	0.448 ± 0.126 ^a^	0.031 ± 0.005 ^b^
Fructose	0.285 ± 0.181 ^a^	0.056 ± 0.065 ^b^	ND
Fumaric acid	0.005 ± 0.002 ^a^	0.004 ± 0.001 ^a^	0.003 ± 0.002 ^a^
Hypoxanthine	0.425 ± 0.158 ^b^	0.761 ± 0.119 ^a^	0.636 ± 0.156 ^ab^
Isobutyric acid	ND	0.102 ± 0.040 ^a^	0.025 ± 0.009 ^b^
Isovaleric acid	0.004 ± 0.001 ^b^	0.107 ± 0.044 ^a^	0.046 ± 0.014 ^b^
Sucrose	0.216 ± 0.217 ^a^	0.085 ± 0.095 ^a^	0.062 ± 0.053 ^a^
Tyramine Xanthine	0.056 ± 0.028 ^b^	0.101 ± 0.038 ^ab^	0.146 ± 0.062 ^a^
Nucleosides	0.008 ± 0.003 ^a^	0.009 ± 0.005 ^a^	0.011 ± 0.003 ^a^
Choline glycerophosphate	0.252 ± 0.067 ^a^	0.357 ± 0.198 ^a^	0.496 ± 0.210 ^a^
*β*-Alanine	0.043 ± 0.015 ^b^	0.195 ± 0.051 ^a^	0.259 ± 0.062 ^a^

^a,b^: different letters indicate significant differences in different groups (*p* < 0.05). ND: none detected.

## Data Availability

The original contributions presented in this study are included in the article/Supplementary Materials. Further inquiries can be directed to the corresponding author.

## Supplementary Materials

The following supporting information can be downloaded at: https://www.mdpi.com/article/10.3390/foods15030459/s1, Table S1: Overall sensory evaluation standards of Coppa; Table S2: Taste sensory scoring criteria for coppa.

## References

[1] Li M., Zhang X., Li J., Liu L., Zhu Q., Qu C., Zhang Y., Wang S. (2023). Identification and in silico simulation on inhibitory platelet-activating factor acetyl hydrolase peptides from dry-cured pork coppa. Foods.

[2] Leni G., Rocchetti G., Bertuzzi T., Abate A., Scansani A., Froldi F., Prandini A. (2024). Volatile compounds, gamma-glutamyl-peptides and free amino acids as biomarkers of long-ripened protected designation of origin Coppa Piacentina. Food Chem..

[3] Rutigliano M., Loizzo P., Spadaccino G., Trani A., Tremonte P., Coppola R., Dilucia F., Di Luccia A., la Gatta B. (2023). A proteomic study of “Coppa Piacentina”: A typical Italian dry-cured Salami. Food Res. Int..

[4] Hospital X.F., Hierro E., Arnau J., Carballo J., Aguirre J.S., Gratacós-Cubarsí M., Fernández M. (2017). Effect of nitrate and nitrite on *Listeria* and selected spoilage bacteria inoculated in dry-cured ham. Food Res. Int..

[5] Ravyts F., Vrancken G., D’Hondt K., Vasilopoulos C., Vuyst L.D., Leroy F. (2009). Kinetics of growth and 3-methyl-1-butanol production by meat-borne, coagulase-negative staphylococci in view of sausage fermentation. Int. J. Food Microbiol..

[6] Rebecchi A., Pisacane V., Callegari M.L., Puglisi E., Morelli L. (2015). Ecology of antibiotic resistant coagulase-negative staphylococci isolated from the production chain of a typical Italian salami. Food Control.

[7] Yang P., Zhong G., Yang J., Zhao L., Sun D., Tian Y., Li R., Rong L. (2022). Metagenomic and metabolomic profiling reveals the correlation between the microbiota and flavor compounds and nutrients in fermented sausages. Food Chem..

[8] Hu M., Yu J., Yu J., Pan Y., Ou Y. (2018). Isolation and screening of *Staphylococcus xylosus* P2 from Chinese bacon: A novel starter culture in fermented meat products. Int. J. Food Eng..

[9] Vural Y., Ferriday D., Rogers P.J. (2023). Consumers’ attitudes towards alternatives to conventional meat products: Expectations about taste and satisfaction, and the role of disgust. Appetite.

[10] De Andrade J.C., Nalério E.S., Giongo C., de Barcellos M.D., Ares G., Deliza R. (2018). Consumer sensory and hedonic perception of sheep meat coppa under blind and informed conditions. Meat Sci..

[11] López-Pedrouso M., Pérez-Santaescolástica C., Franco D., Carballo J., Zapata C., Lorenzo J.M. (2019). Molecular insight into taste and aroma of sliced dry-cured ham induced by protein degradation undergone high-pressure conditions. Food Res. Int..

[12] Rocchetti G., Scansani A., Leni G., Sigolo S., Bertuzzi T., Prandini A. (2023). Untargeted metabolomics combined with sensory analysis to evaluate the chemical changes in Coppa Piacentina PDO during different ripening times. Molecules.

[13] Fu J., Sun C., Chang Y., Li S., Zhang Y., Fang Y. (2023). Structure analysis and quality evaluation of plant-based meat analogs. J. Texture Stud..

[14] Zhou C.Y., Bai Y., Wang C., Li C.B., Xu X.L., Pan D.D., Cao J.X., Zhou G.H. (2021). ^1^H NMR-based metabolomics and sensory evaluation characterize taste substances of Jinhua ham with traditional and modern processing procedures. Food Control.

[15] Yang Y., Pan D., Sun Y., Wang Y., Xu F., Cao J. (2019). ^1^H NMR-based metabolomics profiling and taste of stewed pork-hock in soy sauce. Food Res. Int..

[16] Lou X., Ye Y., Wang Y., Sun Y., Pan D., Cao J. (2018). Effect of high-pressure treatment on taste and metabolite profiles of ducks with two different vinasse-curing processes. Food Res. Int..

[17] Zhang X., Yang J., Gao H., Zhao Y., Wang J., Wang S. (2020). Substituting sodium by various metal ions affects the cathepsins activity and proteolysis in dry-cured pork butts. Meat Sci..

[18] Xu C., Wang Y., Pan D., Zhou C., He J., Cao J. (2021). Effect of cooking temperature on texture and flavour binding of braised sauce porcine skin. Int. J. Food Sci. Technol..

[19] Yao X., Teng W., Wang J., Wang Y., Zhang Y., Cao J. (2024). Polyglycerol polyricinoleate and lecithin stabilized water in oil nanoemulsions for sugaring Beijing roast duck: Preparation, stability mechanisms and color improvement. Food Chem..

[20] (2012). Sensory Analysis—General Guidance for the Selection, Training and Monitoring of Assessors.

[21] (2012). Sensory Analysis—General Guidelines for the Selection, Training and Monitoring of Selected Assessors and Expert Sensory Assessors.

[22] (2004). Sensory Analysis—Methodology—Triangle Test, Spanish.

[23] Ferreira N.B.M., Rodrigues M.I., Cristianini M. (2023). Effect of high pressure processing and water activity on pressure resistant spoilage lactic acid bacteria (*Latilactobacillus sakei*) in a ready-to-eat meat emulsion model. Int. J. Food Microbiol..

[24] De Marins A.R., De Campos T.A.F., Batista A.F.P., Correa V.G., Peralta R.M., Mikcha J.M.G., Gomes R.G., Feihrmann A.C. (2022). Effect of the addition of encapsulated *Lactiplantibacillus plantarum* Lp-115, *Bifidobacterium animalis* spp. Cooked lactis Bb-12 and *Lactobacillus acidophilus* La-5 for hamburger. LWT-Food Sci. Technol..

[25] Gotterup J., Olsen K., Knøchel S., Tjener K., Stahnke L.H., Møller J.K.S. (2008). Colour formation in fermented sausages by meat-associated staphylococci with different nitrite- and nitrate-reductase activities. Meat Sci..

[26] Tanase R., Senda R., Matsunaga Y., Narukawa M. (2022). Taste characteristics of various amino acid derivatives. J. Nutr. Sci. Vitaminol..

[27] Zhang L., Duan W., Huang Y., Zhang Y., Sun B., Pu D., Tang Y., Liu C. (2020). Sensory taste properties of chicken (Hy-Line brown) soup as prepared with five different parts of the chicken. Int. J. Food Prop..

[28] Bogdanov S., Kilchenmann V., Fluri P., Buhler U., Lavanchy P. (1999). Influence of organic acids and components of essential oils on honey taste. Am. Bee J..

