# Supplementary material for: Research on Physicochemical Properties and Taste of Coppa Influenced by Inoculation with Staphylococcus During Air-Drying Process
Source: Foods. 2026 Jan 28;15(3):459. doi: 10.3390/foods15030459 (PMC12896817; doi:10.3390/foods15030459)
Supplement: Supplementary file 1 [file foods-15-00459-s001.zip › foods-4098148-supplementary.pdf]

Supplementary Table S1 Overall sensory evaluation standards of Coppa

Scoring items	Scoring criteria	Definition of sensory intensity (10 points
Color	The cross-section is lustrous, bright red or rose red, and the fat is white	7 ~ 10
	The cross-section is lustrous, with the muscle being grayish red and the fat slightly yellowish	4 ~ 6
	The cross-section has a dull luster, the muscle is dark grey, and the fat is yellowish	1 ~ 3
Texture	The organization is dense and has a good sense of hardness	7 ~ 10
	The organization is relatively tight and the hardness is average	4 ~ 6
	The tissue is loose and has a poor sense of hardness	1 ~ 3
Smell	Rich fermentation aroma	7 ~ 10
	The fermented aroma is relatively mild	4 ~ 6
	The fermentation aroma is insufficient or there is an unpleasant smell	1 ~ 3
Taste	It is neither too salty nor too bland, with a mild sourness and abundant richness	7 ~ 10
	The saltiness is moderate, the sourness is mild, and the richness is sufficient. It is a bit salty or a bit light, the sourness is plain, and the richness is average	4 ~ 6
	Too salty or too bland, with a sour and pungent taste or an odd smell, and poor richness	1 ~ 3
Juiciness	Very good. Easy to chew	7 ~ 10
	General	4 ~ 6
	Poor quality, difficulty in chewing	1 ~ 3
Total score	The total score = color + texture + aroma + taste + juiciness	

Supplementary Table S2 Taste sensory scoring criteria for coppa

Scoring items	Scoring criteria	Definition of sensory intensity (10 points
Saltiness	A score of 0 to 10 indicates an increasing degree of saltiness from low to high	Add 1.50 g of sodium chloride for every 100 mL
Umami	A score of 0 to 10 indicates an increasing degree of umami from low to high	Add 1.00 g of sodium glutamate for every 100 mL
Sweetness	A score of 0 to 10 indicates an increasing degree of sweet taste from low to high	Add 5.00 g of sucrose for every 100 mL
Bitterness	A score of 0 to 10 indicates an increasing degree of bitter taste from low to high	Add 0.08 g of caffeine for every 100 mL
Sourness	A score of 0 to 10 indicates an increasing degree of sour taste from low to high	Add 0.08 g of citric acid for every 100 mL
After-taste	A score of 0 to 10 indicates an increasing degree of aftertaste from low to high	The taste left after chewing in the mouth
Overall-taste	A score of 0 to 10 indicates an increasing degree of saltiness from low to high	
